# Optimized Aluminum Hydroxide Adsorption–Precipitation for Improved Viral Detection in Wastewater

**DOI:** 10.3390/ijerph22020148

**Published:** 2025-01-23

**Authors:** Karla Farmer-Diaz, Makeda Matthew-Bernard, Sonia Cheetham, Kerry Mitchell, Calum N. L. Macpherson, Maria E. Ramos-Nino

**Affiliations:** 1 Department of Microbiology, Immunology, and Pharmacology, School of Medicine, St. George’s University, St George P.O Box 7, Grenada; kfarmer1@sgu.edu (K.F.-D.); mmatthew@sgu.edu (M.M.-B.); 2 Department of Pathobiology, School of Veterinary Medicine, St. George’s University, St George P.O Box 7, Grenada; scheetha@sgu.edu; 3 Department of Public Health and Preventive Medicine, School of Medicine, St. George’s University, St George P.O Box 7, Grenada; kmitche3@sgu.edu; 4 School of Graduate Studies, St. George’s University, St George P.O Box 7, Grenada; cmacpherson@sgu.edu

**Keywords:** wastewater, wastewater-based epidemiology, viral concentrations, aluminum concentration, non-enveloped viruses, enveloped viruses

## Abstract

Wastewater-based epidemiology (WBE) is a valuable tool for monitoring pathogen spread in communities; however, current protocols mainly target non-enveloped viruses. This study addresses the need for standardized methods to detect both enveloped and non-enveloped viruses by testing four aluminum hydroxide adsorption–precipitation techniques. Wastewater samples were spiked with an enveloped virus surrogate (Φ6 bacteriophage) and a non-enveloped virus surrogate (MS2 coliphage), and viral recovery was assessed using reverse-transcription quantitative PCR (RT-qPCR). The highest recovery for the enveloped virus was achieved with AlCl_3_ at pH 3.5, a 15 min flocculation time, and a 3% elution solution concentration. For the non-enveloped virus, optimal recovery was found with AlCl_3_ at pH 6.0, no flocculation time, and a 10% elution solution. The best method for recovering both virus types used AlCl_3_ at pH 6.0, 15 min flocculation, and a 3% elution solution concentration. This study shows that while optimal conditions vary between virus types, a standardized AlCl_3_ flocculation protocol can efficiently recover both, providing a cost-effective approach for outbreak monitoring in Grenada.

## 1. Introduction

Wastewater-based epidemiology (WBE) offers a rapid and cost-effective alternative to individual testing for monitoring community-level pathogen spread [1,2,3]. Developed by Daughton in 2001 [4], the concept of wastewater-based epidemiology (WBE) is based on the idea that any substance excreted by humans and remaining stable in wastewater can be detected and quantified to estimate its original excretion levels [5]. Wastewater acts as a pooled sample of the community that is being monitored, as it comprises biomarkers that have been shed via the population’s urine, feces, saliva, and other bodily secretions [6]. Since viruses cannot replicate outside of a host cell, the quantity of human viruses detected in wastewater reflects the concentration excreted by the population [7,8]. This principle was effectively demonstrated by the global polio eradication program, which used wastewater analysis to monitor and track polio virus levels [9,10,11,12,13]. Additionally, enteric virus [14,15] and hepatitis A levels [16,17] were also monitored by WBE in several communities.

Severe acute respiratory syndrome coronavirus 2 (SARS-CoV-2) is the etiological agent responsible for the deadly Corona Virus Disease-2019 (COVID-19) [18]. Furthermore, during the COVID-19 pandemic, SARS-CoV-2 RNA (Ribonucleic acid) was detected in wastewater from patients’ urine [19] and feces [20], confirming WBE’s potential to reflect virus concentrations excreted by a population [9,10,12,13,21].

Wastewater surveillance has become a valuable tool for monitoring viral pathogens within communities, offering insights into disease prevalence, transmission dynamics, and trends over time [22,23]. This surveillance method is relatively low-cost compared to individual clinical testing, especially clinical molecular testing [1,24,25]. Furthermore, this can provide an early warning system to the public health sector, encompassing individuals who run the gamut from healthy individuals, asymptomatic individuals, and those who require medical intervention [26,27]. Additionally, utilizing WBE data reduces privacy concerns and the need for patients to self-report symptoms, as individual patient data information would not be collected.

A key challenge in wastewater-based epidemiology (WBE) is the development of an efficient concentration method to recover both enveloped viruses, like SARS-CoV-2, and non-enveloped viruses, like norovirus [28,29,30,31,32]. This is especially important since viral concentrations in wastewater are generally lower than those found in clinical samples [32,33]. Although various methods exist for virus concentration in wastewater, most are tailored to non-enveloped enteric viruses [34].

This study was conducted at a pump station in a university setting in Grenada, West Indies, and utilized coagulation and flocculation to concentrate viruses from 24 h composite samples of wastewater This process separates wastewater into liquids and solids, either naturally or enhanced by coagulants that promote particle aggregation [35]. In this study, the aluminum hydroxide adsorption–precipitation method, widely used in wastewater-based epidemiology, was used to concentrate viruses from wastewater samples. This method involved the addition of aluminum chloride (AlCl_3_) to the wastewater, adjusting the pH (typically between 3.5 and 6.0), which causes aluminum hydroxide to precipitate and form positively charged flocs. These flocs attract and adsorb viral particles, which are generally negatively charged at neutral pH. During the process, the sample is mixed to promote adsorption, and the flocs are separated by centrifugation. The resulting pellet, which contains concentrated viral particles, can be resuspended in a small volume of elution buffer—often a beef extract solution—to release the viruses for further analysis [36,37].

The aim of this study was to develop a singular protocol that can be utilized for the recovery of both enveloped and non-enveloped viruses from wastewater in an environment with low resources. The aluminum hydroxide adsorption–precipitation method is valued for its simplicity, low cost, and adaptability, making it suitable for detecting a range of enveloped and non-enveloped viruses in wastewater. Studies have shown that AlCl_3_ effectively recovers SARS-CoV-2 [38], suggesting flocculation can be a promising approach for concentrating various virus types. Enhancing wastewater surveillance tools through methods like flocculation could provide a rapid, cost-effective alternative to clinical testing for monitoring public health threats in the future.

## 2. Materials and Methods

### 2.1. Sample Collection and Concentration Methods

Four independent concentration methods were evaluated on six composite wastewater samples collected from September to October 2023. Twenty-four-hour composite samples were gathered using an autosampler, collected at 30 min intervals, refrigerated, and transported to the lab for analysis.

Inside a BSL-2 safety cabinet, 50 mL aliquots from each composite sample were used for four concentration experiments. Each method involved spiking wastewater with 1 × 10^6^ plaque-forming units per milliliter (PFU/mL) of *Pseudomonas syringae* phage Φ6 (an enveloped, double-stranded RNA virus surrogate to SARS-CoV-2) and the non-enveloped coliphage MS2. Pepper Mild Mottle Virus (PMMoV), a highly abundant and stable virus, served as an internal control to confirm the presence of human fecal content in each sample.

One milliliter of each spiked sample was collected before treatment, and RNA was directly extracted using the TRIzol™ phenol–chloroform method as per the manufacturer’s recommendations (ThermoScientific), which isolates RNA despite potential PCR inhibitors like proteins, fats, carbohydrates, polyphenols, metal ions, and RNAses in wastewater [39,40]. The extracted RNA was then analyzed by RT-qPCR to evaluate the effectiveness of the RNA concentration protocols.

Method 1 employed an aluminum-driven flocculation method modified from the Standard Practice for Recovery of Viruses from Wastewater Sludges and Peppa and Gerba [41,42,43]. Fifty milliliters of wastewater was acidified to pH 3.5 using AlCl_3_ and spiked with *Pseudomonas syringae* phage Φ6 (enveloped virus) and MS2 (non-enveloped virus) at 12.5 µL PFU/mL. The sample was centrifuged at 3300× *g* for 10 min, then the supernatant was discarded, and the pellet was resuspended in 2 mL of 10% beef extract (Oxoid Hampshire, Basingstoke, England). After further centrifugation at 1700× *g* for 20 min, the supernatant was filtered through a 0.22 µm filter (Corning^®^ 150 mL Vacuum Filter/Storage Bottle System, 0.22 µm Pore CA Membrane, Corning, San Diego, CA, USA) and retained for RNA extraction and RT-qPCR analysis.

Method 2, modified from Randazzo et al. [44,45], adjusted 50 mL of wastewater to pH 6.0 with a 1:100 ratio of 0.9 N AlCl_3_. The sample was spiked with both *Pseudomonas syringae* phage Φ6 and MS2, agitated at room temperature for 15 min, and then centrifuged at 1700× *g* for 20 min. The pellet was resuspended in 2 mL of 3% beef extract (Oxoid Hampshire, Basingstoke, England), shaken at 150 rpm for 10 min, and centrifuged at 1900× *g* for 30 min. The concentrate was resuspended in 1 mL of 0.01 M Phosphate-Buffer Saline (PBS) for RNA extraction and RT-qPCR analysis.

Method 3 combined elements from Method 1 and Method 2 as 50 mL wastewater was acidified to pH 3.5 with AlCl_3_, spiking with both *Pseudomonas syringae* phage Φ6 and MS2, and agitating at room temperature for 15 min. The sample was centrifuged at 1700× *g* for 20 min, and the pellet resuspended in 3% beef extract (Oxoid Hampshire, Basingstoke, England), shaken, and centrifuged to form a concentrate, then resuspended in 1 mL 0.01 M PBS for RNA extraction and RT-qPCR analysis.

Method 4 also combined techniques from Method 1 and Method 2, adjusting 50 mL wastewater to pH 6.0 with AlCl_3_. The sample was spiked with *Pseudomonas syringae* phage Φ6 and MS2 at 12.5 µL PFU/mL. The sample was centrifuged at 3300× *g* for 10 min, then the supernatant was discarded, and the pellet was resuspended in 2 mL of 10% beef extract (Oxoid Hampshire, Basingstoke, England). After further centrifugation at 1700× *g* for 20 min, the supernatant was filtered through a 0.22 µm filter (Corning^®^ 150 mL Vacuum Filter/Storage Bottle System, 0.22 µm Pore CA Membrane, Corning, San Diego, CA, USA) and retained for RNA extraction and RT-qPCR analysis.

### 2.2. RNA Extraction and Analysis

Viral RNA was extracted using the TRIzol™ method as recommended by the manufacturer. The purity and concentration of the RNA obtained were determined through 260/280 nm absorbance measures using the NanoDrop spectrophotometer 2000 (Thermo Scientific, Waltham, MA, USA). RT-qPCR was performed using Taqman technologies (IDT Integrated DNA technology) for the presence of *Pseudomonas syringae* phage Φ6 [46], MS2 [47], and PMMoV [48] using TaqMan™ Fast Virus 1-Step Master Mix (Thermo-Fisher Scientific) in a 20 µL volume reaction. Samples were run in duplicates with corresponding positive (standards) and negative controls (DNase-and RNase-free water) on an AriaMx Real-Time PCR system (Agilent Technologies, Inc., Santa Clara, CA, USA). The thermal cycling conditions of the qPCR assays were as follows: reverse transcription at 50 °C for 5 min, then 95 °C for 20 s, followed by 42 cycles of 3 s at 95 °C and 30 s at 55 s. Previously established standard curves available in our laboratory with an R2 ≥ 0.99 with amplification efficiencies of 90–110% were used to quantify the spiked and recovered viruses. Nuclease-free water (Invitrogen, Waltham, MA, USA) was used as a negative control for each test.

The recovery efficiencies were determined by comparing the concentrations of the spiked *Pseudomonas syringae* phage Φ6 and MS2 before and after flocculation. All viral concentration experiments for each method were conducted in duplicate for this study and evaluated based on the average Ct reduction values (Ct of non-flocculated sample-Ct of flocculated sample) as previously described in [49].

### 2.3. Statistical Analysis

Data normality was assessed using the Shapiro–Wilk test. A one-way analysis of variance (ANOVA) was used to compare the recovery methods. The independent and combined effects of the methods and the surrogate viruses on Ct reduction were analyzed using a two-way ANOVA. When statistical significance was observed, post hoc analysis was conducted using Tukey’s HSD for multiple comparisons (α = 0.05; 95% confidence interval). All analyses were performed using RStudio (version 2024.04.2 Build 764).

## 3. Results

Based on the intra-replicate standard deviation of Ct values obtained (Table 1), the tested protocols demonstrated good reproducibility in concentrating the viral surrogates (*Pseudomonas syringae* phage Φ6 and MS2) and the internal control, PMMoV, from the wastewater samples collected.

As observed in Table 2, except for *Pseudomonas syringae* phage Φ6 in Method 1, the average Ct values for the enveloped (*Pseudomonas syringae* phage Φ6) and non-enveloped viruses (MS2) were recovered better than the internal control PMMoV, which served as an indicator of each method’s concentration efficiency or internal control.

**Figure 1:**
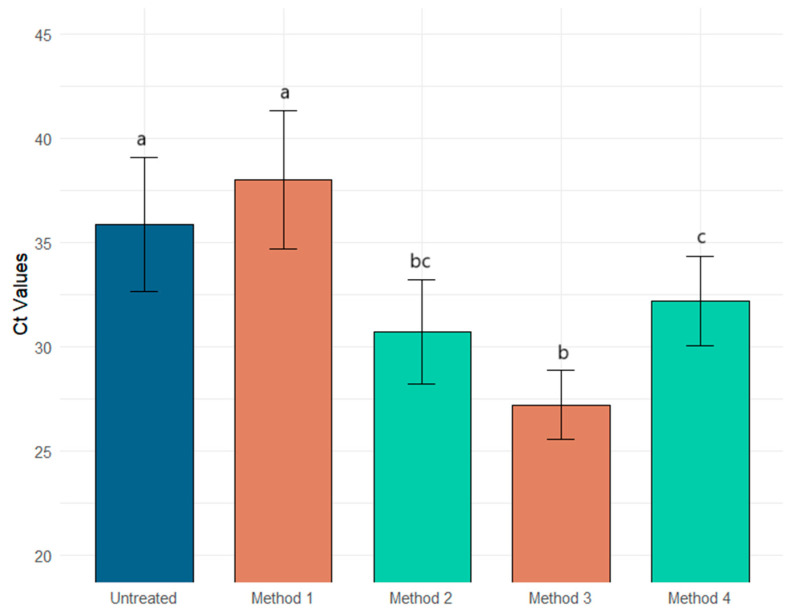
Comparison of the four AlCl_3_ methods reveals statistically significant higher recovery (indicated by lower Ct values) from Methods 2, 3, and 4 for the enveloped surrogate compared to the untreated. Different letters between groups indicate statistical significance. Blue: untreated; orange: pH 3.5; and green: pH 6.0.

**Figure 2:**
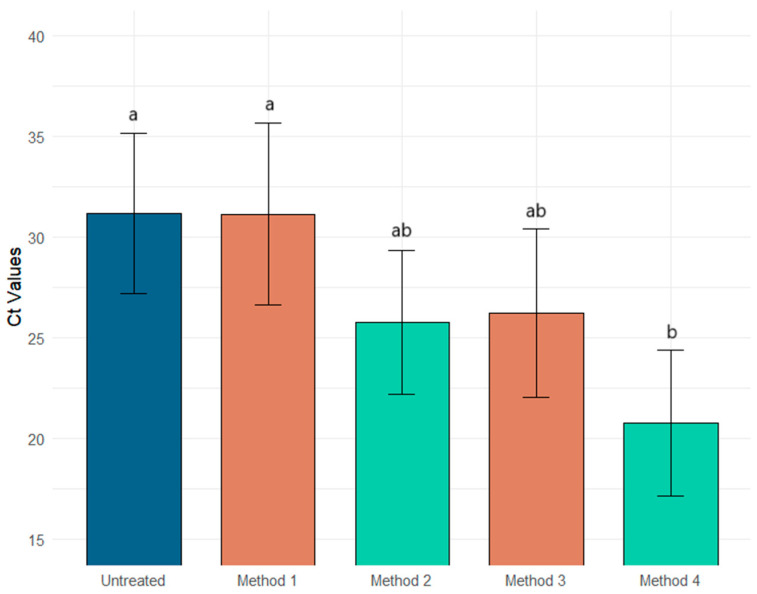
Comparison of the four AlCl_3_ methods reveals significantly higher recovery (indicated by lower Ct values) from Method 4 for the non-enveloped surrogate compared to the untreated. Different letters between groups indicate statistical significance. Blue: untreated; orange: pH 3.5; and green: pH 6.0.

The Ct reduction by method and virus type is presented in Table 3. Method 1 was the least effective in concentrating the enveloped and non-enveloped viral surrogates, resulting in almost no Ct reductions for the Φ6 and MS2 assays with −2.14 ± 4.27 [CI 95% −5.56–1.28] and 0.04 ± 5.34 [CI 95% −4.22–4.31] reductions, respectively. Method 2 had a similar concentration reduction for both surrogates, with a reduction of 5.17 ± 4.47 [CI 95% 1.59, 8.75] and 5.41 ± 2.98 [CI 95% 3.03, 7.79], respectively. The better recovery rate for the enveloped surrogate was observed for Method 3, 8.6 ± 3.67 [CI 95% 5.74, 11.61] and for the non-enveloped virus surrogate, Method 4, 10.41 ± 5.96 [CI 95% 5.64, 15.18]. Figure 1 and Figure 2 show the relationship between the Ct values of enveloped and non-enveloped surrogates compared to the untreated samples.

**Figure 3:**
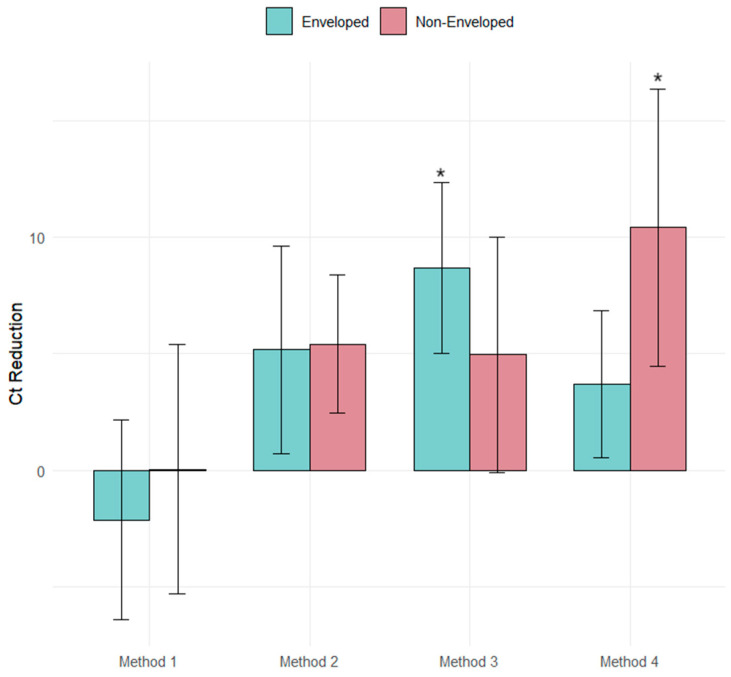
Overall Ct reduction comparison. Method 1 showed the smallest Ct reduction. Method 4 used with non-enveloped viruses showed a statistically significant larger Ct reduction and Method 3 used with enveloped viruses showed a statistically significant larger Ct reduction in an overall comparison. * 95% significance level.

There is clear evidence of an interaction between the method used and the surrogate used on Ct reduction. Method 3 is the most effective method for recovering enveloped viruses (Figure 1), while Method 4 is the best for non-enveloped viruses (Figure 2). Although the interaction is not statistically significant, Method 2 is the most effective for recovering both enveloped and non-enveloped viruses (Figure 3).

The effect of the pH alone can be observed in the comparison between Methods 1 vs. 4 and Methods 2 vs. 3 since they have different pH values but the same secondary processes (flocculation time, and elution solution concentration).

**Figure 4:**
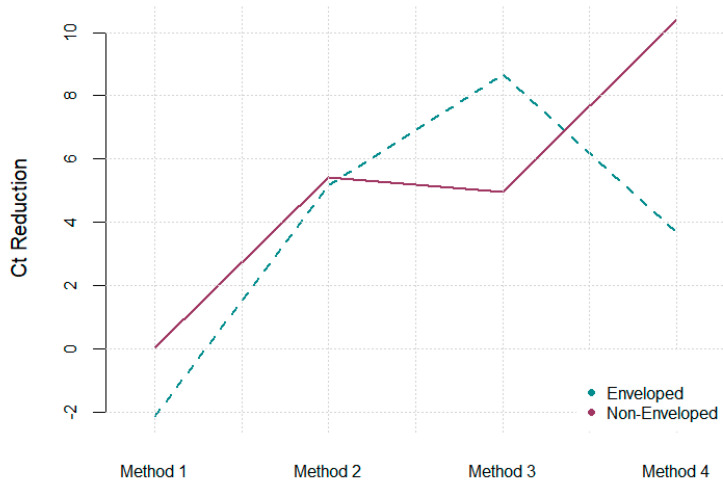
Interaction plot showing Ct reduction across different methods and surrogates demonstrating that Method 4 is the best recovery method for non-enveloped viruses, Method 3 for enveloped viruses, and Method 2 the best combination method for enveloped and non-enveloped viruses.

Methods 1 (pH = 3.5) and 4 (pH = 6) with flocculation time of zero minutes and elution solution concentration of 10% beef extract show higher but no significant difference at pH = 6 between the enveloped and enveloped comparison (*p* adj = 0.34), and significant difference between non-enveloped and non-enveloped comparison (*p* adj = 0.006), as well as between enveloped and non-enveloped comparison (*p* adj = 0.0005), showing the importance of pH for the recovery of viruses under these conditions (Figure 4).

Methods 2 (pH = 6) and 3 (pH = 3) with 15 min flocculation time and elution solution concentration of 3% beef extract show higher but no significant difference at pH = 3 for the enveloped to enveloped comparison (*p* adj = 0.87); but no difference for the enveloped to non-enveloped comparison (*p* adj = 1.00) or non-enveloped to non-enveloped comparison (*p* adj = 1.00), suggesting that pH is less critical under these conditions.

## 4. Discussion and Conclusions

The Aluminum Hydroxide Adsorption–Precipitation method is a technique for concentrating viruses, bacteria, and other pathogens from wastewater or environmental samples, particularly in wastewater-based epidemiology (WBE). This method exploits the ability of aluminum hydroxide (Al(OH)_3_) to adsorb viral particles and aggregate them into larger flocs, which can then be separated from the liquid phase. Floc formation is a result of coagulation, where fine particles clump together to form larger particles (flocs) due to the use of chemicals (coagulants) [52]. Aluminum is used as a coagulant because it can form multi-charged polynuclear complexes, enhancing its ability to adsorb particles [53]. When added to water, aluminum chloride reacts to form aluminum hydroxide precipitates. These precipitates, formed through the hydrolysis of Al^3+^ ions in water, attract and trap negatively charged viral particles, effectively concentrating them for further analysis [54,55].

In this study, we looked at several factors that may influence viral recovery with the use of aluminum hydroxide including different combinations of pH, composition and concentration of the elution buffer, flocculation time, and viral type.

The pH of the wastewater sample plays a critical role in forming aluminum hydroxide precipitates. The pH affects the hydrolysis of Al^3+^ ions and the charge of the flocs, influencing their ability to adsorb viral particles. At pH 6, many viruses carry a slight negative charge due to their isoelectric points. When pH is slightly acidic, surfaces like the aluminum hydroxide flocs (from AlCl_3_) develop a positive charge. This charge differential encourages electrostatic interactions, enhancing virus adsorption onto the coagulant or membrane [43].

Furthermore, many viruses are more stable under slightly acidic conditions than at highly alkaline pH levels, which helps preserve their integrity during the concentration and recovery process; however, some respiratory viruses demonstrated decreased viability under a pH of 3 [56]. Too low or too high a pH can hinder floc formation, as coagulation is generally most effective in the pH range of 5.7–8.0 [57].

In our study, pH 6 (Methods 2 and 4) showed the formation of flocs that effectively capture virus particles, but only significant differences with other methods could be observed for non-enveloped viruses. Recent wastewater epidemiology studies support these findings, particularly for SARS-CoV-2 [38,58,59]. These studies, as reviewed in various publications on virus concentration methods, underscore the practical benefits of pH 6 in wastewater-based surveillance and public health monitoring.

Another parameter that could affect virus recovery with this method is the concentration of the elution buffer. Viruses are typically recovered after retention on filter membranes by eluting them from the collection substrate [41]. For filter membranes, as those used in Method 1 and 4, a common elution solution used is an alkaline mixture of beef extract [60]. When passed through the filter, this solution promotes virus desorption by restoring the virus’s natural negative charge in an alkaline environment. This charge reversion increases the repulsion between the viruses and the membrane, facilitating their release and improving recovery efficiency.

In this study, the non-enveloped virus recovered better at pH 6 and higher beef extract concentration (10%) (Method 4). Beef extract elution is probably more effective for recovering the non-enveloped viruses than enveloped viruses due to structural differences that affect how each virus interacts with the elution solution. Non-enveloped viruses, like enteric viruses, have a durable protein capsid that is less prone to damage from the components in beef extract, such as proteins, peptides, and amino acids. Enveloped viruses like SARS-CoV-2 possess a lipid envelope surrounding their capsid. This lipid membrane is more vulnerable to the alkaline conditions of beef extract solutions, which may destabilize the envelope, leading to potential inactivation or incomplete recovery.

Flocculation time is also an important parameter for recovering viruses. Floc formation involves the aggregation of viruses and other suspended particles, often aided by chemicals (coagulants), like aluminum chloride (AlCl_3_). An adequate period is necessary for these aggregates to develop to a size suitable for efficient removal via sedimentation or filtration. If the time allotted for floc formation is too short, viral particles may not fully adhere to the flocs, resulting in lower recovery rates. Additionally, compounds present in wastewater, like humic acid and proteins, may enhance the formation of aluminum hydroxide precipitates [61]. Also, a short time for coagulation formation may lead to smaller floc sizes, which may not settle or filter out effectively, allowing viruses to remain in the liquid phase.

Research has shown that the optimal flocculation period can vary based on several factors, including the type of virus, the characteristics of the wastewater, and the specific coagulants used [38,58,62,63]. In our study, a longer floc formation increased the recovery of enveloped viruses in combination with pH = 3.5 and 3% beef extract eluent concentration. Further research is needed to investigate the independent effect of the elution buffer and the flocculation time on the optimal recovery of enveloped and non-enveloped viruses with this method.

This is the first study that we know of and has been conducted in the Caribbean using wastewater-based epidemiology for the recovery of viral pathogens. This method for viral recovery can be an important initiative for policymakers and stakeholders in the Caribbean region as it can provide timely and actionable information to mitigate disease outbreaks, especially in cases of natural disasters (like hurricanes).

## Figures and Tables

**Table 1:** Intra-replicate standard deviation of Ct values obtained for each of the four concentration protocols and the three viruses PMMoV (internal control), *Pseudomonas syringae* phage Φ6 (enveloped virus), and MS2 (non-enveloped virus).

Methods	Ct PMMOV	Intra-Replicate Standard Deviation	Ct Φ6	Intra-Replicate Standard Deviation	Ct MS2	Intra-Replicate Standard Deviation
Method 1	29.53	0.74	41.01	4.09	37.00	1.98
	29.71		41.01		37.00	
	28.23		33.75		35.41	
	28.47		34.12		32.80	
Method 2	28.50	0.96	26.74	3.81	28.48	3.07
	28.81		26.94		32.93	
	27.21		26.33		29.66	
	26.83		34.28		35.20	
Method 3	29.50	0.50	28.29	0.66	23.75	0.78
	29.69		28.97		24.17	
	30.36		27.80		22.56	
	30.52		27.46		22.74	
Method 4	27.88	1.62	30.99	0.24	15.98	0.68
	31.81		31.33		15.60	
	29.51		31.56		15.38	
	29.54		31.37		16.92	

**Table 2:** Average Ct reduction values for all concentration methods vs. untreated samples on the three viruses PMMoV (internal control), *Pseudomonas syringae* phage Φ6 (enveloped virus), and MS2 (non-enveloped virus).

Methods	Target		PMMOV	Φ6	MS2
	Ct cut off value		>40 *	>41	≥37 **
Untreated wastewater	Spiked wastewater Ct	Sample 1	36.90	39.64	36.84
		Sample 2	39.69	33.54	28.20
		Sample 3	38.13	34.57	27.04
		Sample 4	40.15	35.33	30.52
		Sample 5	39.02	39.97	35.23
		Sample 6	38.17	32.29	29.27
	Average Ct		38.68	35.89	31.18
Method 1	Spiked wastewater AFTER flocculation Ct	Sample 1	28.99	37.47	35.55
		Sample 2	29.03	32.00	37.00
		Sample 3	37.12	41.01	25.72
		Sample 4	39.34	40.90	27.96
		Sample 5	35.08	38.40	28.41
		Sample 6	35.11	38.27	32.18
	Average Ct		34.11	38.02	31.14
Method 2	Spiked wastewater AFTER flocculation	Sample 1	27.89	28.57	31.57
		Sample 2	29.91	29.17	27.74
		Sample 3	33.07	31.53	21.36
		Sample 4	35.98	35.44	23.28
		Sample 5	32.85	29.80	25.89
		Sample 6	34.67	29.81	24.80
	Average Ct		32.38	30.72	25.77
Method 3	Spiked wastewater AFTER flocculation	Sample 1	30.02	28.13	23.31
		Sample 2	30.76	30.01	19.99
		Sample 3	32.14	25.18	27.30
		Sample 4	29.62	26.59	27.10
		Sample 5	34.15	26.58	32.31
		Sample 6	34.82	26.78	27.36
	Average Ct		31.92	27.21	26.23
Method 4	Spiked wastewater AFTER flocculation	Sample 1	29.69	31.31	15.97
		Sample 2	34.43	32.79	16.41
		Sample 3	37.42	31.40	23.05
		Sample 4	36.97	29.58	21.81
		Sample 5	39.38	35.84	23.91
		Sample 6	40.01	32.40	23.48
	Average Ct reduction		36.32	32.20	20.77

* [50], ** [51].

**Table 3:** Viral concentration reduction achieved by each of the four protocols expressed as Ct reduction, along with the associated time required per protocol. Initial wastewater volume (50 mL) to final volume wastewater (2 mL).

Methods	ΔCt PMMOV	CI	ΔCt Φ6	CI	ΔCt MS2	CI	Time (Hours)
Method 1	4.53	1.41, 7.72	−2.14	−5.56, 1.28	0.04	−4.22, 4.31	3.5
Method 2	6.29	3.91, 8.32	5.17	1.59, 8.75	5.41	3.03, 7.79	4
Method 3	6.76	4.65, 8.87	8.67	5.74, 11.61	3.69	0.92, 8.99	3.5
Method 4	2.36	−0.69, 2.88	4.95	1.16, 6.22	10.41	5.64, 15.18	4

## Data Availability

All data are provided in the article.
